# Quantitative proteomic analysis of serum-purified exosomes identifies putative pre-eclampsia-associated biomarkers

**DOI:** 10.1186/s12014-022-09342-4

**Published:** 2022-02-10

**Authors:** Rosana Navajas, Antonio Ramos-Fernandez, Ignacio Herraiz, Alberto Galindo, José Luis Bartha, Fernando Corrales, Alberto Paradela

**Affiliations:** 1 Functional Proteomics Facility, Centro Nacional de Biotecnología (CNB-CSIC), ProteoRed-ISCIII, Madrid, Spain; 2 Proteobotics, Madrid, Spain; 3 Fetal Medicine Unit, Maternal and Child Health and Development Network (SAMID), Department of Obstetrics and Gynecology, Hospital Universitario 12 de Octubre, Instituto de Investigación Hospital 12 de Octubre, Madrid, Spain; 4 Division of Obstetrics and Maternal Fetal Medicine, La Paz University Hospital, Madrid, Spain

**Keywords:** Biomarkers, Cohort studies, Exosome, Mass spectrometry, Plasma, Pre-eclampsia, Pregnancy, Proteomics, Serum

## Abstract

**Background:**

The high incidence of pre-eclampsia, which affects 2–7% of all pregnancies, remains a major health concern. Detection of pre-eclampsia before the appearance of clinical symptoms is essential to allow early intervention, and would benefit from identification of plasma/serum biomarkers to help guide diagnosis and treatment. Liquid biopsy has emerged as a promising source of protein biomarkers that circumvents some of the inherent challenges of proteome-wide analysis of plasma/serum. In this respect, purified exosomes have the added benefit of being carriers of intercellular communication both in physiological and pathological conditions.

**Methods:**

We compared the protein complement of purified exosomes from three different collections of control and pre-eclamptic serum samples, obtained at the end of the second trimester of pregnancy and at delivery. We employed shotgun label-free proteomics to investigate differential protein expression, which was then validated by targeted proteomics.

**Results:**

We developed a purification method that yielded highly enriched exosome preparations. The presence of specific pregnancy protein markers suggested that a significant proportion of purified exosomes derived from tissues related to pregnancy. Quantitative proteomic analyses allowed us to identify 10, 114 and 98 differentially-regulated proteins in the three sample collections, with a high degree of concordance. Functional analysis suggested that these proteins participate in biological processes related to pre-eclampsia, including angiogenesis, inflammation and cell migration. The differential abundance of 66 proteins was validated by targeted proteomics. Finally, we studied the impact of the pre-eclampsia-associated exosomes in the proteome using an in vitro cellular model.

**Conclusions:**

We have identified and validated differential exosomal proteins in liquid biopsy of pregnant women that open new possibilities for early detection of pre-eclampsia. Additionally, the functional impact of the proteome composition of purified pre-eclamptic exosomes in target cells provides new information to better understand changes in embryo-maternal interactions and, consequently, the pathogenesis of this disease.

**Supplementary Information:**

The online version contains supplementary material available at 10.1186/s12014-022-09342-4.

## Introduction

Pre-eclampsia is a leading cause of fetal and maternal morbidity and mortality, affecting 2–7% of all pregnancies and accounting for 40% of fetal deaths worldwide [1]. Concurrent new-onset hypertension (systolic blood pressure ≥ 140 mm Hg, diastolic blood pressure ≥ 90 mm Hg) and proteinuria (≥ 0.3 g in 24-h collected urine sample or a spot urine protein:creatinine ratio ≥ 30 mg/mmol) are the most characteristic clinical manifestations of the disease, and swelling usually appears between weeks 20–34 (early-onset pre-eclampsia, EOPE) or later (late-onset pre-eclampsia). Other less prevalent symptoms include intense headaches or visual disturbances, acute renal failure, epigastric pain, liver injury, pulmonary edema, thrombocytopenia and hemolysis. If left untreated, pre-eclampsia can progress to convulsions/seizures (eclampsia) in the most severe cases [2]. Population studies have clearly established that pre-eclampsia negatively affects the health of both the mother and the child later in life––for instance, women are prone to develop diabetes, hypertension, coronary artery disease and stroke, and children can present with an increased risk for metabolic and cardiovascular diseases [2–4].

Important advances made in our understanding of the pathogenesis of pre-eclampsia have focused on the evident impaired placental function brought about by disturbances in trophoblast invasion and spiral-artery remodeling [2]. While it is well established that key regulators of the angiogenic balance that modulate early placental vascular development and trophoblast invasion are dysregulated in pre-eclampsia, there is no consensus on the main triggering factors of the disease and no preventive strategy has yet been identified. A placenta-released soluble form (sFlt-1) of the vascular endothelial growth factor receptor (VEGFR1, Flt-1) has been reported to be upregulated in patients with pre-eclampsia [5]. sFlt-1 can bind both VEGF and its homolog placental growth factor (PlGF), preventing their engagement with membrane-bound Flt-1 and causing soluble VEGF/PlGF levels to diminish. Binding of both VEGF and PlGF to Flt-1/VEGFR is essential for embryonic angiogenesis (neovascularization) and vasculogenesis (de novo vessel formation) [6, 7]. Another protein found overexpressed in pre-eclampsia is soluble endoglin (sEng) [8], which further contributes to the dysregulation of angiogenic balance by preventing the interaction of TGF-β1 to its receptor in the vasculature [9], thus also functioning as an antiangionic protein. These proteins are currently used as diagnostic biomarkers of the disease [10, 11], and calculation of the sFlt-1/PlGF ratio is considered to reflect alterations in the angiogenic balance.

There is a lack of consensus about cut-off values, gestational age for screening and other important parameters for pre-eclampsia that has fueled the search for new biomarkers for either diagnosis or prognosis [12, 13]. A biomarker commonly refers to a molecule of biological origin that is a measurable indicator of the molecular mechanisms implicated in normal or disease processes and, potentially, could be used for diagnostic/prognostic purposes [14, 15]. Omics technologies, focusing on the massive and unbiased detection and quantification of genes (genomics), mRNAs (transcriptomics), proteins (proteomics) and metabolites (metabolomics) have become the ideal platform for biomarker discovery [16]. However, the complexity of pre-eclampsia in terms of maternal predisposition, genetic inheritance and associations with several medical conditions (diabetes mellitus, chronic hypertension and metabolic syndrome, among others) that confer increased risk, reflect the huge number of putative protein biomarkers described to date [15, 17].

Within the area of disease-associated biomarker discovery, circulating extracellular vesicles, and more specifically exosomes, are now in focus as a novel liquid biopsy approach in disease diagnosis. Exosomes are a specific subtype of extracellular vesicle defined by their endosomal origin (the presence of endosomal markers such as Tsg101, CD9, CD63, and CD81) and their size range (50–150 nm) and density (1.13–1.19 g/mL) [18]. As mediators of intercellular communication, exosomes are specifically packaged with a wide array of cargo molecules (e.g., proteins, lipids, messenger RNAs, microRNAs and noncoding RNAs) that reflect their cellular and tissue origin [18], and are released into extracellular biofluids by exocytosis to transmit biological signals. Placental cells and tissues release exosomes, both in vitro and in vivo*,* and the abundance of placenta-derived exosomes in maternal blood seems to increase steadily during pregnancy, particularly during the first trimester [19]. Microparticle shedding from syncytiotrophoblasts has been described to be higher in pre-eclampsia than in normal pregnancy [20]. Exosomes have been investigated in many biology and disease models and are recognized to play critical roles in angiogenesis, immunomodulation, cell survival, cancer and inflammation; however, their importance in mediating embryo-maternal interactions, implantation, placental physiology and pregnancy has only recently been appreciated [21]. Recent evidence has demonstrated that exosomes purified from women who have normal or pre-eclamptic pregnancies elicit different responses related to vascular functions in in vitro models of endothelial function [22–24] and the low oxygen tension present in pre-eclamptic placenta [25]. Likewise, cell migration and proliferation in a trophoblast cell model was impaired by the presence of a key microRNA (miR-125a-5p) in pre-eclampsia-derived exosomes [26]. In the same line, the upregulation of placenta-associated serum exosomal mir-155 from patients with pre-eclampsia may suppress endothelial nitric oxide synthase (eNOS) expression and nitric oxide (NO) production in endothelial cells [27]. In a similar context, the administration of human umbilical cord mesenchymal stem cell-derived exosomes was protective in an in vivo rat model of eNOS-induced pre-eclampsia [28].

An environment of immune tolerance during pregnancy is necessary to prevent rejection of the fetus, which can be considered as a semi-allogeneic graft for the mother. Several regulatory mechanisms contribute to this process, including altered antigen presentation, T cell differentiation and proliferation and activation of regulatory cells [29], and it has been shown that proteins present in exosomes, such as syncitin-1, can contribute to fetomaternal immunotolerance [30]. Immune-suppressive ligands on exosomal membranes (e.g., FasL, TRAIL, NKG2D and PDL-1) have been described to regulate apoptosis and cytotoxicity of maternal leucocytes [31]. Th1 and Th2 cytokine profiles seem also to differ when comparing exosomes from normal and pre-eclamptic pregnancies [32].

These data, overall, suggest an active role of extracellular vesicles, and more specifically exosomes, in the communication between the fetus, the placenta and the mother [29], facilitated by the delivery of cargo molecules modulating important physiological processes involved in maintenance of immunotolerance, angiogenesis, implantation, migration and/or cell proliferation. Changes in exosomal cargo would, accordingly, have a direct effect on these physiological processes, as suggested by the differential effects induced by exosomes of normotensive and pre-eclamptic origin.

In the present study, we compared the proteomes of molecular size exclusion-purified serum exosomes obtained from three independent sample collections of normal and pre-eclamptic pregnancies. We obtained clinical samples either at the end of the second trimester or at delivery and we performed an exhaustive proteomic characterization of the exosome population to identify potential pre-eclampsia-associated biomarkers. In addition to the prognostic and diagnostic interest of protein biomarkers, they might also shed new light on the molecular mechanisms implicated in this disease. In this context, functional experiments carried out on an in vitro cell-culture model demonstrated that the addition of purified exosomes disturbs the quantitative composition of the targeted cell proteome.

## Materials and methods

### Sample collection

Serum samples (Additional file 3: Table S1) were independently obtained from the Department of Obstetrics and Gynecology (sample collection C1 from Hospital La Paz, Madrid) and from IDIBAPS Biobank (sample collections C2 and C3, Hospital Clinic, Barcelona). Samples correspond to sera previously collected from age-matched normal pregnant women (controls) and patients with EOPE (diagnosed before 34 weeks of gestation) and were obtained after informed consent following the ethical guidelines of the institutions. Samples from subjects (control or pre-eclamptic) affected by another pathology or receiving any kind of treatment were excluded from the analysis. Sample collection C1 included 4 controls and 4 cases of EOPE, taken at delivery. Sample collection C2 and C3 included 10 control sera and 10 cases of EOPE independently collected at two different gestational periods (25–27 weeks and at delivery for sample collections C2 and C3, respectively). An additional sample collection (C4, from Hospital 12 de Octubre, Madrid) comprised 11 control and 11 cases of EOPE obtained at 26–32 weeks, and was used as a validation cohort for targeted proteomics. Diagnosis of EOPE followed the guidelines established by the International Society for the Study of Hypertension in Pregnancy [33]. Serum samples were immediately stored at – 80 °C before exosome isolation.

### Exosome isolation by size exclusion chromatography

Isolation of serum exosomes (small extracellular vesicles, sEVs) was established using the size-based classification system recommended by the International Society of Extracellular Vesicles [34] with size exclusion chromatography (SEC) (qEV, Izon), as described [35]. Two centrifugation cycles at 1500×*g* and 10,000×*g* (4 °C) for 10 and 20 min, respectively, were first applied to 500 μL of serum to remove large extracellular vesicles. The qEV column was rinsed with 10–15 mL of PBS (pH 7.4), which was filtered (0.2-μm sterile syringe filter, PALL) and degassed for 10 min beforehand using an ultrasonic bath. After column equilibration, the sample was applied and 20 fractions of 0.5 mL were collected. The first 6 fractions (3 mL), corresponding to the column void volume, were discarded, and the fractions containing sEVs (eluting in fractions 7–9, 1.5 mL) were pooled and saved. Most serum proteins eluted in fractions 10–40 and were also discarded. Pooled sEV-containing fractions (1.5 mL) were either concentrated to a final volume of 30 μL using a Speed Vac (sample collection C1 and C4), or were concentrated using Nanosep centrifugal ultrafiltration devices (Omega, cut-off 10 kDa, PALL, serum sample collections C2 and C3), to reduce volumes with minimal protein loss. In the latter case, the membrane was pre-treated with 5% sodium dodecyl sulfate (SDS) followed by washing with water and centrifugation at 14,000×*g*, room temperature, for 5 min. Subsequently, the sample was loaded onto the device, which was centrifuged again at 14,000×*g*, room temperature, in several cycles of 10 min until a residual volume containing the vesicles was achieved. For efficient collection of the sEV-containing extract, 50 μL of RIPA lysis buffer was added directly onto the device (see lysis procedure below).

### Exosome characterization based on size, morphology, and concentration

sEVs from a pool of serum samples (n = 7) were enriched by SEC and characterized using transmission electron microscopy (TEM) and nanoparticle tracking analysis (NTA). Both techniques are recommended to assess the quality and efficacy of sEV isolation. Size distribution and concentration of the isolated sEVs was determined on a NanoSight LM 10 instrument equipped with an LM14 laser module, syringe pump system and a CCD camera (Malvern Instruments, Malvern, UK). The sample (2 μL) was diluted in 1 mL of PBS (1:500, v/v) to give an optimal range of particle concentration of 1 × 10^8^–1 × 10^9^ particles/mL. The following settings were used: camera level 11, detection threshold 2 and acquisition time 30 s. Data analysis was performed with NTA v3.1 software. Samples were analyzed in triplicate, and the final size distribution and particle concentration was the average of the three measures. TEM analysis was used for size and morphology characterization of enriched vesicles. The sample (2 μL) was diluted in 8 μL of PBS (1:4, v/v) and stained with 2% (w/v) uranyl acetate. Negative staining of sEVs was examined with a JEOL JEM 1011 (JEOL, Peabody, MA) transmission electron microscope equipped with a Gatan ES1000Ww camera, applying resolutions of 30,000 and 60,000.

### Recovery and purity of exosomes

Exosome extraction efficiency involves both sEV recovery and purity, so that both parameters are correlated. We measured the particle number to evaluate the isolation efficiency in terms of the sEV yield. The NTA provided a particle number/mL of 4.76 × 10^8^ (in a 2-μL aliquot), corresponding to 3.57 × 10^11^ total particles in the 1.5-mL sEV fraction. To assess the purity of the exosomal preparations, we measured the amount of protein (5–20 μg in the different control/EOPE samples) to obtain protein yield. Sample purity was calculated as particle number to protein amount ratio (sEV number/μg of protein) [36]. Assuming that ratios greater than 3 × 10^10^ have been proposed as high purity isolates, 12 μg would be the sample purity threshold (sample protein amounts greater than 12 μg would suggest low purity samples). To further assess the purity, we used tandem mass spectrometry-based identification of exosomal protein markers included in a reference list extracted from Exocarta and EVpedia repositories [37]. The sample acceptance criterion was the identification of a minimum number of exosomal marker proteins included in the reference list, and only samples with at least 9 exosome marker proteins (corresponding to 25% of the list) were accepted for subsequent quantitative analysis. This quality criterion fully agrees with the sEV number/μg of protein ratios. Thus, control (C1 and C8) and EOPE (PE2 and PE6) samples from sample collection C2 (gestational week 25–27) all with less than 6 exosomal markers and protein amount greater than 20 μg, were excluded. Likewise, control C8 and PE9 samples from sample collection C3 (at delivery) with 8 and 4 exosome markers, respectively, and with protein amount of 18 and 23 μg, were also excluded. Excluded samples would be poor in exosomes and highly contaminated with soluble serum proteins.

### Differential proteomics analysis by label free quantification

Label-free quantitative proteomics analysis was carried out following a bottom-up strategy, with 4 or 10 biological replicates in each condition, depending on the sEV sample.

### Lysis of exosomal proteins

sEVs from the Speed Vac evaporator (30 μL) or Nanosep ultrafiltration device were lysed with 100 μL or 50 μL, respectively, of RIPA lysis buffer (150 mM NaCl, 1% (v/v) Triton X-100, 0.5% (w/v) sodium deoxycholate, 0.1% (w/v) SDS, 50 mM Tris–Cl), containing protease inhibitors (Complete Mini, EDTA-free, Roche, Basel, Switzerland). The procedure consisted of two incubations, at 95ºC for 10 min and 5 min on ice, followed by sonication (4 × 10 s/cycle), and incubation on ice for 5 min. Subsequently, a centrifugation step at 12,000×*g* and 4 °C for 10 min was performed, and the supernatant was saved.

### Protein precipitation, quantification, and in-solution digestion (sample collections C1 and C4)

sEV protein lysates were precipitated with methanol/chloroform. Four volumes of methanol were added to 1 volume (typically 100 μL) of sample dissolved in aqueous buffer, plus 1 volume of chloroform and 3 volumes of water; the mixture was vortexed and centrifuged at 15,000×*g* at 10 °C for 10 min. The aqueous top layer was then carefully discarded without removing the interface layer, and 4 volumes of methanol were added to the remaining volume. The mixture was vortexed and centrifuged again at 15,000×*g* at 10 °C for 10 min. The liquid was removed (as much as possible) and the protein pellet was dried in a laminar flow cabinet and stored at – 20 °C. Protein quantitation was performed using the Pierce 660-nm Assay. Protein extracts were resuspended in 7 M urea, 2 M thiourea, 100 mM triethylammonium bicarbonate (TEAB), cysteines were reduced in 5 mM Tris (2-carboxyethyl)phosphine (TCEP) at 37 °C for 1 h and alkylated in 20 mM methyl methanethiosulfonate (MMTS) at room temperature for 10 min. After adjusting the TEAB concentration to 25 mM, trypsin was added (enzyme-to-protein ratio of 1:20), and proteolysis was allowed to proceed at 37 °C overnight. All reagents were purchased from Sigma-Aldrich (Madrid, Spain).

### S-trap in-column digestion and quantification by QuBit (sample collections C2 and C3)

We used S-trap microcolumns (PROTIFI) to improve digestion yields from low-abundant samples difficult to solubilize. SDS and TEAB were added to 5–20 μg of samples at a final concentration of 5% (w/v) and 50 mM, respectively. Cysteine reduction and alkylation was performed as described above, followed by addition of 12% phosphoric acid to a final phosphoric acid: sample ratio of 1:10 (v:v). After sample acidification, binding buffer solution (BBS; 90% methanol, 100 mM TEAB, pH 7) was added to give a final BBS:sample ratio of 6:1 (v:v), and the mixture was loaded onto the S-trap column in several fractions. Each sample aliquot was centrifuged at 3000×*g* at room temperature for 2 min, and the flow-through was discarded. After 3 additional washing cycles with BBS, in-column proteolysis with trypsin in 100 mM TEAB (enzyme to protein ratio 1:10) was performed overnight at 37 °C. Peptide elution was carried out by the sequential addition of 40 μL of 25 mM TEAB, 25 μL of 0.2% formic acid and 35 μL of 0.2% formic acid in 50% acetonitrile, followed by centrifugation (3000×*g*, room temperature, 2 min). Fractions were combined, dried in a Speed Vac and stored at – 20 °C. Quantification of the peptide digests was performed at the peptide level using the QuBit platform (Invitrogen).

### Tandem mass spectrometry analysis by LC–MS/MS

One-microgram aliquots of tryptic peptides per sample (injection volume 5 μL) were analyzed on a nano liquid chromatography system (Eksigent Technologies nanoLC Ultra 1D plus, AB SCIEX, Foster City, CA) coupled to a 5600 Triple TOF mass spectrometer (AB SCIEX) with a nanoelectrospray ion source. Samples were injected into a C18 PepMap trap column (5 µm, 100 µm I.D. × 2 cm, Thermo Scientific) at 2 µL/min, in 0.1% formic acid in water, and the trap column was connected on-line to a C18 nanoAcquity BEH analytical column (1.7 µm, 100 Å, 75 µm I.D. × 15 cm, Waters). The nanopump provided a flow-rate of 250nL/min and the gradient elution conditions were as follows: 0.1% formic acid in water as mobile phase A, and 0.1% formic acid in acetonitrile as mobile phase B, from 5 to 40% B in 120 min. The mass spectrometer operated in data-dependent acquisition mode. For MS1 scans, the accumulation time was set to 250 ms and up to 10 precursor ions were acquired per spectrum (100 ms for each MS2), which represents a total cycle time of 1.3 s. This shorter duty cycle allows for the acquisition of a greater number of points per MS1 ion precursor, which is essential to improve quantitation in label-free based quantitative approaches.

### Shotgun proteomics data analysis

Raw MS spectra were converted to mgf format using Peak View v1.2.0.3 and searched using Mascot v2.6.1, OMSSA 2.1.9, X!TANDEM Alanine 2017.2.1.4 and Myrimatch 2.2.140 against a composite target/decoy database built from the human sequence reference proteome downloaded from Uniprot Knowledgebase (https://www.uniprot.org/proteomes/). The database contained 74,788 *Homo sapiens* sequences (last modified on November 5, 2019) plus a short list of common contaminants (keratins and proteases). All searches were run from a command-line interface except in the case of Mascot, which was operated using Mascot Server/Daemon. Search engines were configured to match potential peptide candidates with a precursor mass error tolerance and MS2 fragment ion tolerance of 25 ppm and 0.02 Da, respectively. Up to two missed tryptic cleavage sites and a maximum isotope error (^13^C) of 1 were allowed. Beta-methylthiolation of cysteine and oxidation of methionine, possible pyroglutamic acid from glutamine or glutamic acid at the peptide N-terminus and acetylation of the protein N-terminus were considered as fixed and variable modifications, respectively. Score distribution models were used to compute peptide-spectrum match p-values [38] and to generate standardized meta-scores to enable the integration of search results from multiple search tools. The false discovery rate (FDR) associated with each observed meta score was estimated from the spectra of a balanced holdout set of identified peptides using tenfold cross-validation. The cross-validated FDR was used as a ranking variable in the four-engine ensemble, and only peptides recovered with a peptide-level FDR ≤ 0.01 filter were selected for protein inference and quantitative analysis. Within each protein group, the smallest set of parent proteins accounting for all identified peptides was obtained by extensive enumeration. Label-free quantification was based on simultaneous modeling of isotopic envelopes and elution profiles of clusters of co-eluting peptides to obtain robust peak area estimators. Differential regulation was measured using linear models [39] on log2 ratios built from equivalent signals in each pair of samples. Groups of shared peptides within protein groups were treated as separate quantitative hypotheses. Contrast p-values were estimated specifically for each protein by simulation from random effects models. Statistical significance was measured using q-values (FDR). All analyses were conducted using software from Proteobotics (Madrid, Spain).

### Targeted proteomics analysis

One microgram of sample (in a 5-μL volume) was analyzed on an Eksigent 1D Plus nanoHPLC coupled to a 5500 QTRAP triple quadrupole mass spectrometer (AB SCIEX). Analogous mobile phases and the trapping column as described above were used, but with the following modifications: C18 BioSphere analytical column (3 µm, 120 Å, 75 µm I.D. × 15 cm, Nanoseparations), a nanoflow of 300 nL/min, a loading pump flow of 3 μL/min and 60-min gradient elution conditions (0–42 min from 2 to 40% B). Multiple reaction monitoring (MRM) analysis mode was applied for the acquisition of 3–4 transitions per proteotypic peptide. Likewise, their equivalent standard synthetic peptides spiked into a background matrix of an *Escherichia coli* tryptic digest were analyzed in parallel. Skyline software package (v4.2.0) was used for the selection of proteotypic peptides and transitions, for acquisition method development and, finally, for the analysis of the results [40]. Declustering potential was 80 V and collision energy was automatically calculated as a function of the peptide length. Dwell times of 20–25 ms per transition (duty cycle of 2.8–3.5 s) were used for C1 samples. For sample collections C2 and C3, where the number of peptides and transitions to monitor was too high, several sub-methods (4–5) were developed. After data analysis and filtering of doubtful or undetectable peptides, scheduled methods (1–2) were performed. Specific details about peptide parental ions and transitions m/z are provided in Additional file 4: Table S2.

### Targeted proteomics data analysis

Monitored proteotypic peptides were validated by comparison of their transition intensity profiles and retention times with those of standard synthetic peptides. For the analysis of quantitative differences between EOPE and control samples, weighted sums of log-transformed transition intensities were used as protein abundance estimators for subsequent statistical analyses. Weights were inverse variances estimated by fitting an intensity-dependent exponentially-decreasing residual variance trend. After normalization using a linear model, EOPE *versus* control contrast p-values for each protein were computed from ANOVA F-tests, and significance was determined by controlling the FDR [41]. Centered and scaled protein abundance values were used for principal component analysis (PCA) as an unsupervised method.

### BeWo and HUVEC cell cultures

#### Culture conditions

The human placenta choriocarcinoma cell line BeWo (ATCC CCL-98) was cultured in F-12 K base medium and 10% fetal calf serum (FCS), according to the ATCC (American Type Culture Collection) recommendations. To prevent bacterial contamination, penicillin–streptomycin was added at a final concentration of 10 mL/L. Human endothelial cells (HUVECs, ATCC CRL-1730) were cultured in the same complete growth medium (F-12 K, 10% FCS) with penicillin–streptomycin, plus 0.1 mg/mL of heparin and 1% (v/v) of endothelial cell growth supplement, following ATCC recommendations. Both cell types are adherent and were incubated in standard conditions (5% carbon dioxide, 37 °C) in 6-well plates (Corning). Cells were subcultured when they reached 80–90% confluence. BeWo cells were directly detached from the plate by gentle pipetting, whereas HUVECs required incubation for 4–5 min with 0.25% (w/v) trypsin/0.53 mM ethylenediaminetetraacetic acid (EDTA) solution before detachment. Detached cells were collected by centrifugation at 800×*g* at room temperature and were re-seeded.

#### Proteomics study on the interaction of serum exosomal isolates of control/pre-eclampsia samples with BeWo cultures

sEVs enriched from serum samples were added to cultured BeWo cells for different time periods (0, 12 and 24 h) using 4 replicates per condition. Before (4 h) the addition of serum sEVs to BeWo cells, the growth medium was replaced with complete growth medium supplemented with 10% exosome-free FCS, which was achieved by ultrafiltration using Nanosep Omega columns (Pall). An equal number of cells per well (75,000–100,000, equivalent to 10–12 µg of protein) were seeded in 48-well plates (Corning) and incubated for 24 h and, subsequently, 2 µg of SEC-isolated serum sEVs from control/EOPE samples taken at delivery, were added to each well. For each incubation time and control/EOPE replicate, cells were detached, transferred to Eppendorf tubes and collected by centrifugation at 200×*g* (room temperature). The cellular pellet was resuspended in 10 µL of 2 × loading buffer (4% (w/v) sodium dodecyl sulfate, 10% (w/v) 2-mercaptoethanol, 20% (v/v) glycerol, 0.004% (w/v) bromophenol blue, 0.125 M Tris–HCl) and 1 µL of protease inhibitors. Samples were stored at − 20 °C.

#### Differential proteomics analysis of BeWo or HUVECs by label-free quantification

One microliter of nuclease (ThermoFisher) (10 min, room temperature) was added to BeWo or HUVEC cellular extracts obtained as described above. Samples were denatured at 95ºC for 5 min, and were then concentrated by electrophoresis (12% SDS-PAGE). Runs were carried out at 20 mA/gel until samples entered into the resolving area of the gel. Gels were stained with Coomassie Blue (Quick Coomassie, Generon) for 30 min. A one-third fraction of each lane was excised, covering the entire range of protein molecular weights, and subjected to automatic in-gel digestion. Each gel fraction was excised into small pieces, deposited in a 96-well plate, and automatically processed in a Proteineer DP digestor (Bruker Daltonics, Bremen, Germany). The digestion protocol was based on that described by Schevchenko et al. [42] with minor modifications. Proteomics-grade trypsin (Sigma-Aldrich) was added at a final concentration of 16 ng/μl in 25% acetonitrile/50 mM ammonium bicarbonate solution, and samples were incubated at 37 °C for 4 h. Peptides were recovered in 50% acetonitrile/1% trifluroacetic acid, dried in a Speed Vac, and stored at − 20 °C.

#### Tandem mass spectrometry by LC–MS/MS and data analysis

Samples (1 µg) were analyzed by LC–MS/MS analysis as described above but a longer gradient was used (from 5 to 60% B in 200 min). Label-free quantification and proteomic data analyses were performed as described earlier.

## Results

### Exosome isolation and characterization

We purified sEVs using a commercial SEC-based method (Izon), with some modifications [35] (Additional file 1: Fig. S1). The average size of purified exosomes was 168 nm (mode, 123 nm), as determined by NTA, and the yield obtained from an initial volume of 500 µL of serum was 5–20 µg of total protein, equivalent to 3.57 × 10^11^ particles. These values are mostly consistent with those of other authors [43]. The relatively low yield described for SEC-based methods is compensated by greater exosome enrichment due to lower co-isolation of contaminating serum proteins. In the case of sample collections C2 and C3, sEVs were first concentrated by ultrafiltration, and were then lysed and tryptically digested using S-TRAP columns. Tryptic peptides were analyzed by shotgun proteomics (data-dependent acquisition mode) and the MS1 and MS2 spectra were used to launch searches against a human protein database. Results are summarized in Table 1. The total number of identified proteins varied between experiments, and a very significant improvement in terms of total protein identification was evident when Nanosep cartridges and S-Trap columns were used (collections C2 and C3) as compared with standard protein digestion (sample collection C1). The total number of peptides identified (FDR < 1%, calculated at the peptide level) for C1 was 71,355, whereas for C2 and C3 the total numbers were 274,960 and 233,047, respectively. In terms of proteins, 631 were identified in C1, 1557 in C2 and 805 in C3 (Additional file 5: Table S3a, Additional file 6: Table S3b, Additional file 7: Table S3c). The superior results with the Nanosep and S-Trap columns are likely related to reduced protein loss and better sample clean-up. The list of proteins identified from the different sample collections was compared against a reference list of exosome and extracellular vesicle markers, resulting in the identification of a considerable number of exosomal proteins including well-known markers such as CD9, CD63 and CD81 (Table 1) consistent with the quality assessment based on the identification of a minimum number (9 of 34) of characteristic exosomal proteins per sample. Finally, considering that exosomes purified from serum could reflect diverse tissue origins, we searched for pregnancy marker proteins, among which alkaline phosphatase, placental type (ALPP) and pregancy zone protein (PZP) are two of the most relevant. As shown in Table 1, both proteins were detected in all sample collections with a significant number of peptides (Additional file 5: Table S3a, Additional file 6: Table S3b, Additional file 7: Table S3c). Other pregnancy-related proteins such as different isoforms of pregnancy-specific beta-1-glycoprotein and proteins highly expressed in placenta and pregnancy-related tissues were also detected (Additional file 8: Table S4). These results suggest that purified exosomes are secreted by pregnancy-related tissues.

**Table 1:** Summary of the results obtained in the proteomics analysis of sample collections C1–C3

	Sample collection # 1	Sample collection # 2	Sample collection # 3
N# proteins identified^1^	631	1557	805
N# of exosomal proteins^2^	23/34	32/34	29/34
CD9 (P21926)^3^	+^8^	+	+
CD63 (P08962)^4^	n.f.^9^	+	+
CD81 (P60033)^5^	+	+	+
ALPP (P05187)^6^	+	+	+
PZP (P20742)^7^	+	+	+

1: False discovery rate (FDR) ≤ 1% at the peptide level; 2: reference list of exosomal proteins as described in ref. 33; 3: CD9 antigen; 4: CD63 antigen; 5: CD81 antigen; 6: alkaline phosphatase, placental type; 7: pregnancy zone protein; 8: at least one unique peptide identified; 9: not found

### Quantitative proteomics of exosomal proteins

**Fig. 1:**
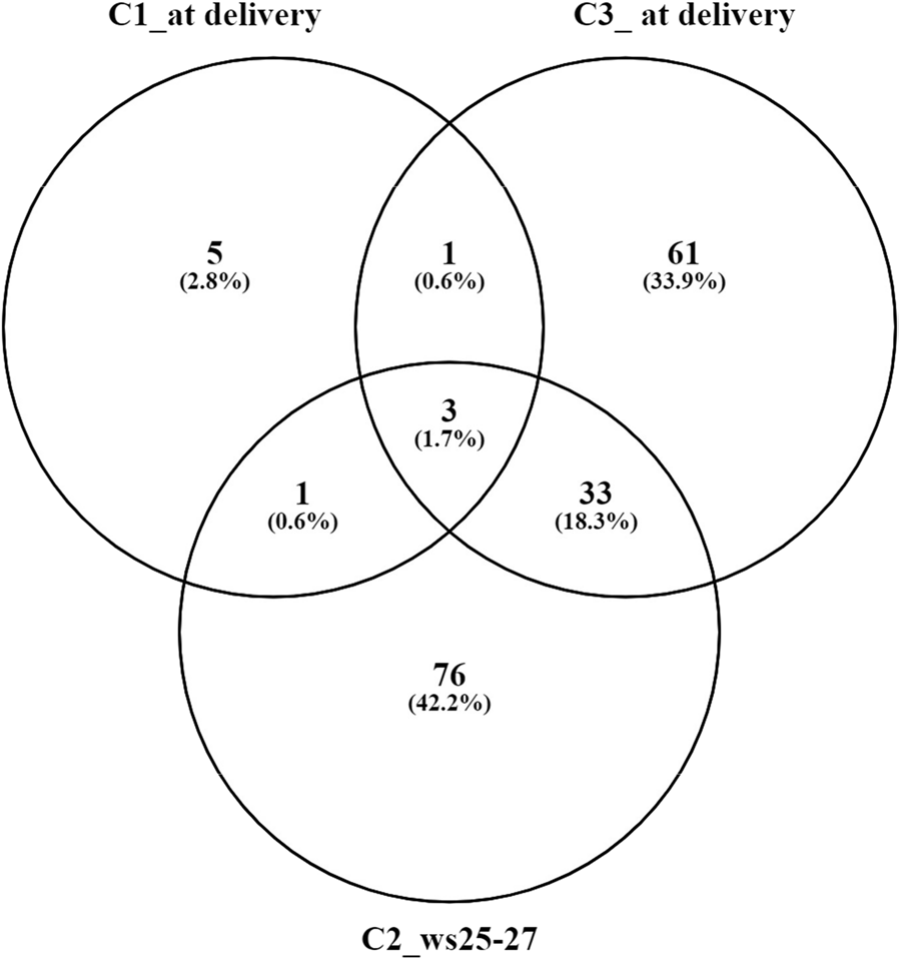
Venn diagram of differentially-regulated proteins with statistical significance (q < 0.1) in the quantitative proteomic analysis of exosomes purified from control and preeclamptic sera in sample collections C1–C3

We used label-free quantitative proteomics to analyze the differences between control and EOPE exosomes in the three independent sample collections, as it is a better approach for the analysis of low-level protein samples (5–20 μg) than approaches based on isobaric labeling (e.g., iTRAQ or TMT). Moreover, label-free quantitative techniques offer greater precision with a lower ratio compression effect, although this comes with a longer instrument time and a higher computational cost in data analysis. In global terms, the number of differentially-regulated proteins (q-value < 0.1) in the different sample collections analyzed followed the pattern described for the identifications. Thus, for sample collection C1, 10 differentially-regulated proteins were identified (q-value < 0.1), while for C2 and C3, this increased to 113 and 98, respectively (Additional file 9: Table S5). Only 3 proteins, PZP, reelin (RELN) and Sushi, von Willebrand factor type A (SVEP1) were differentially expressed in all data sets, possibly due to the differences in the total number of proteins quantified and in the sampling periods (end of the second trimester *versus* delivery). In all cases, PZP and RELN were more abundant in control than in EOPE samples; SVEP1 abundance was also higher in controls obtained at delivery (C1 and C3) but was lower than in EOPE samples from the end of the second trimester (C2). We found a high degree of overlap between the differentially-regulated proteins in the different sample collections despite the differences in the sampling period (Additional file 3: Table S1). Four proteins, peroxiredoxin-2 (PRDX2), SVEP1, RELN and PZP, representing almost 50% of the 10 significantly different proteins quantified in C1 were found in C2 and C3, and an additional protein (versican, VCAN) was found both in C1 and C3. Sample collections C2 and C3 shared 36 proteins corresponding to 30–40% of the statistically-significant differentially-abundant proteins (Fig. 1). With respect to the quantitative results, we also found a high degree of agreement among samples. Thus, the four common proteins (SVEP1, VCAN, RELN and PZP) in the sample collections obtained at delivery (C1 and C3) showed similar quantitative changes (either EOPE < C or EOPE > C) despite being independent studies carried out at different times and with some modifications in the experimental procedure (Additional file 10: Table S6). The comparison between sample collections at different periods of pregnancy revealed a more complex picture. Approximately half of all significant differential proteins (q-value < 0.1) and shared between C2 (week 25–27) and C3 (at delivery) showed a reversal in their ratio (from EOPE > C to EOPE < C and vice versa) as the pregnancy progressed, while the remaining half was unchanged. Notably, shared proteins in inter-collection comparisons from different gestational ages maintained or changed expression in a similar manner. For example, the three common proteins (RELN, PZP, and SVEP1) in C2 (week 25–27) *versus* C1 (at delivery) and C2 (week 25–27) *versus* C3 (at delivery) comparisons showed the same trend (Additional file 10: Table S6), confirming the robustness of the experimental approach. Finally, a functional analysis of the proteins found as positively or negatively regulated was performed to determine their association with EOPE. The analysis showed (Additional file 11: Table S7) that among the 221 proteins described as regulated (including redundancies) in the three sample collections, a very significant number of proteins were involved in biological processes closely related to pre-eclampsia, such as angiogenesis (n = 20 proteins), inflammatory processes (n = 30), immunity (n = 42), migration (n = 4) and proliferation of T cells (n = 7) and linked to the interaction with the extracellular matrix (n = 23). Likewise, biological processes linked to vascular endothelium (n = 15 proteins), blood pressure (n = 5), vasodilation (n = 3) and vasoconstriction (n = 3) were very relevant, as were proteins related to the oxidative stress response (n = 9) and hypoxia (n = 7).

### Targeted proteomics validation

**Fig. 2:**
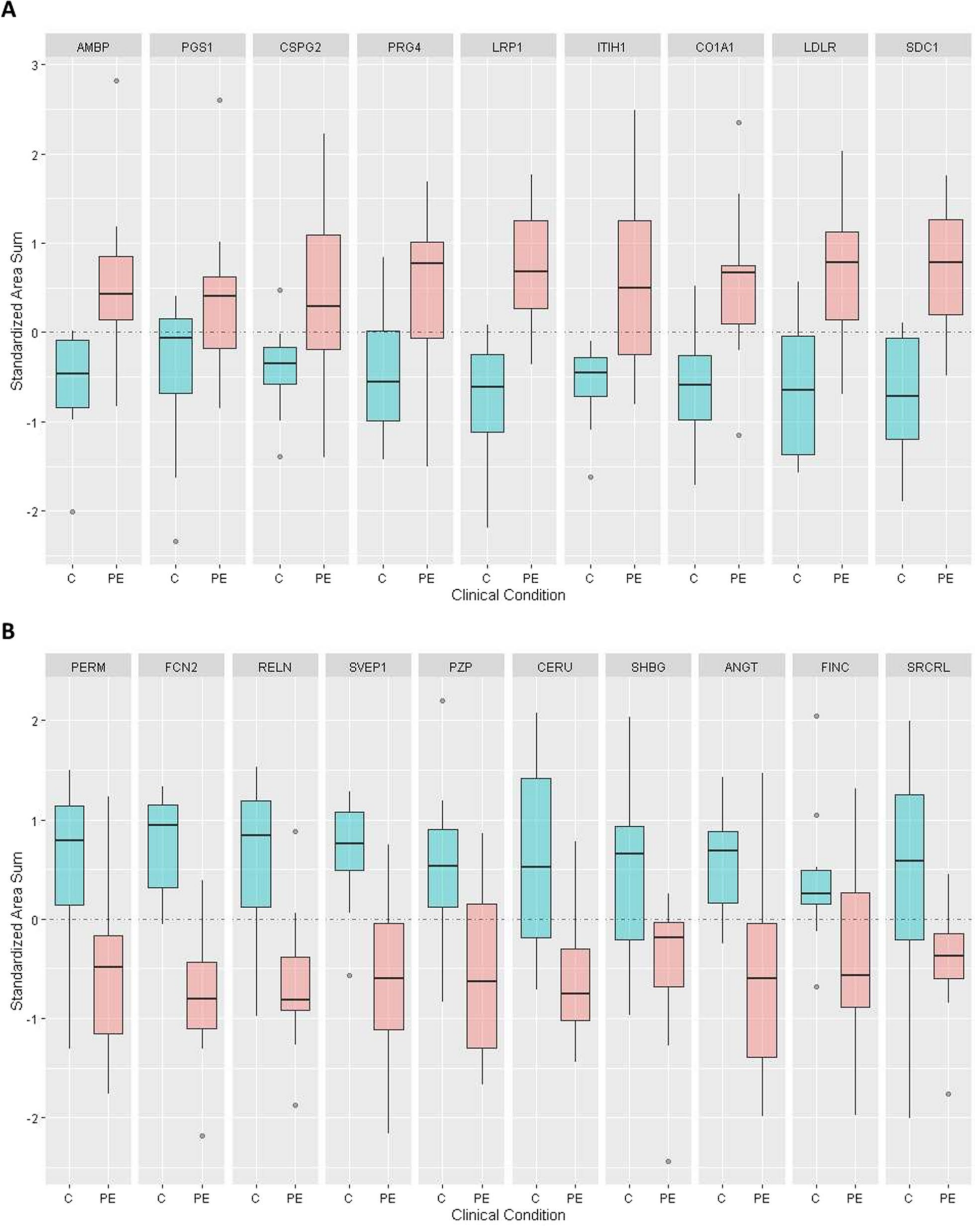
Boxplots of differences in estimated abundance in pre-eclampsia (PE) and control (C) samples. Only proteins with q < 0.1 are displayed. Sums of areas are in standardized scale so that they can be compared across proteins. **A** Results corresponding to up-regulated proteins (EOPE > C) in sample collection C3 (at delivery); **B** results corresponding to down-regulated proteins (EOPE < C) in C3 (at delivery)

The quantitative results were validated by MRM targeted proteomics analysis, in which specific peptides unique to target proteins are monitored through peptide fragments or transitions. Due to the high number of statistically-significant regulated proteins, particularly in sample collections C2 and C3, we applied a series of filters to the initial list to select the best candidates for detection and quantification. In principle, all the proteins detected and quantified with < 4 proteotypic peptides were discarded from the MRM analysis, with the exception of syndecan-1 (SDC1) (C2 and C3), which has previously been reported as exosomal in origin. Syndecan is a low molecular weight protein and, therefore, yields few protetotypic tryptic peptides. Serum abundant proteins were also excluded, as well as those with subtle expression changes (0.3 > log_2_ (EOPE/C) > − 0.3). This resulted in 6 proteins monitored by MRM analysis for sample collection C1, 40 for C2, and 52 for C3, giving a total of 98 proteins including redundancies in the three sample collections. In most cases, the number of specific peptides per protein used for monitoring was 3, although a noticeable number of proteins were monitored by 4–5 peptides. In a few cases, 1 or 2 proteotypic peptides were used. A validation criterion was applied for each experimental peptide based on its specific transition co-elution, as well as on the comparison with its corresponding standard synthetic peptide (spiked-into in an *E. coli* background), with which there should be correspondence in terms of retention time and transition pattern. Several proteins were not detected in any case or experimental evidence was weak (poor peak shape, low signal intensity). Ultimately, the MRM analysis allowed the detection of 78/98 selected proteins. The failure to detect several of the previously detected candidate proteins is not unusual in proteomics and likely reflects the cumulative effect of different experimental variables and especially of the monitoring of proteotypic peptides through precursor ion-fragments (transitions) present with very low intensity in the MS2 spectra. To improve the robustness of the results, 12 proteins verified with a single proteotypic peptide were rejected, with the exception of syndecan-1 (from sample collection C3). The MRM transition list of the remaining 66 proteins is shown in Additional file 4: Table S2. Statistical analysis of the MRM results indicated significant quantitative differences for 39 proteins (q-value < 0.1; Additional file 12: Table S8). In the latter cases, the direction of the regulation (EOPE > C or EOPE < C) confirmed the label-free analysis data, although the numerical values were slightly different, as might be expected from different analytical methods. In some cases, such as for PZP, the prior results suggesting a negative regulation (EOPE < C) of this protein in EOPE *versus* control samples were confirmed in C1 and C3 but not in C2. To clarify this contradictory result, we performed an additional analysis in MRM format, monitoring PZP in an independent sample collection. The result (Additional file 12: Table S8, sample collection from *Hospital 12 de Octubre*) confirmed the statistical significance of the results (EOPE < C) previously obtained. Figure 2 shows characteristic examples of proteins validated by MRM in C3 (see Additional file 2: Fig. S2a–c for C1, C2 and cohort 12 de Octubre). The evident data dispersion is characteristic of quantitative proteomic analysis of clinical samples.

### In vitro cell stimulation assays

Exosomes are released into the extracellular space and transmit biological information to recipient cells [44]. As a functional test, we investigated whether exosomes purified from control/EOPE serum samples could induce changes in the proteome of BeWo cells, a human placental cell line derived from a choriocarcinoma. To do this, we added an equivalent of 2 µg of purified exosomes to 75,000–100,000 BeWo cells in 48-well plates, which were incubated for different times (0, 12 and 24 h). Cells were then detached, washed and processed for proteomic analysis. Quantitative proteomics data revealed that, under the study conditions, exosomes purified from control/EOPE serum induced statistically-significant changes (q < 0.05) in the BeWo cell proteome after 12 h of incubation, but not at time 0 or 24 h. At 12 h, the addition of EOPE serum exosomes increased the expression of 56 proteins relative to cells incubated with control exosomes (Additional file 13: Table S9). Of note, the panel of regulated proteins included proteins involved in physiological processes linked to EOPE such as hypoxia (hypoxia up-regulated protein 1, HYOU1) and protein folding and stabilization (heat shock protein HSP90, HSP90AA1; protein disulfide-isomerase A4, PDIA4; endoplasmic reticulum chaperone BiP, HSPA5). The list also included a relevant number of proteins involved in transcription (e.g., nucleolar RNA helicase 2, DDX21), translation (e.g., eukaryotic translation initiation factor 5B, EIF5B), and replication (e.g., DNA replication licensing factor, MCM4) processes, suggesting that the internalization of exosomes of pre-eclamptic origin activates these processes in target cells. We repeated this experiment using a primary cell line of endothelial origin (HUVEC) as the target cell; however, quantitative proteomic analysis failed to detect any statistically-significant regulated protein (data not shown).

## Discussion

Pre-eclampsia is a hypertensive disorder that affects a very significant percentage of pregnant women and is a major contributor to mortality and morbidity. Moreover, it affects the health of the mother and the fetus both during pregnancy and later in life [1–4] and, accordingly, its prevention is a clinical aspiration. In this line, the search for biomarkers to predict pre-eclampsia, before the appearance of clinical symptoms, will be crucial for the application of preventive therapeutic strategies that minimize the impact of the disease. In addition to improving the accuracy of diagnosis, new biomarkers should also aid in dissecting the biological processes involved, facilitating the design of new and better therapeutic strategies [17]. Blood serum and/or plasma is a very attractive and accessible source of putative biomarkers, including proteins, as it is in close contact with all tissues and organs of the body and remains reasonably stable under standard conditions of conservation [45]. Plasma and/or serum contain and transport proteins and other biomolecules secreted by different organs and tissues of the body, including placenta and other pregnancy-related tissues. However, the analytical potential of these samples as a source of protein biomarkers is limited by their complex composition and the presence of a small panel of extraordinarily abundant proteins (n ≈ 20, representing more than 95% of the protein mass), which considerably complicates experimental methodology [46].

**Fig. 3:**
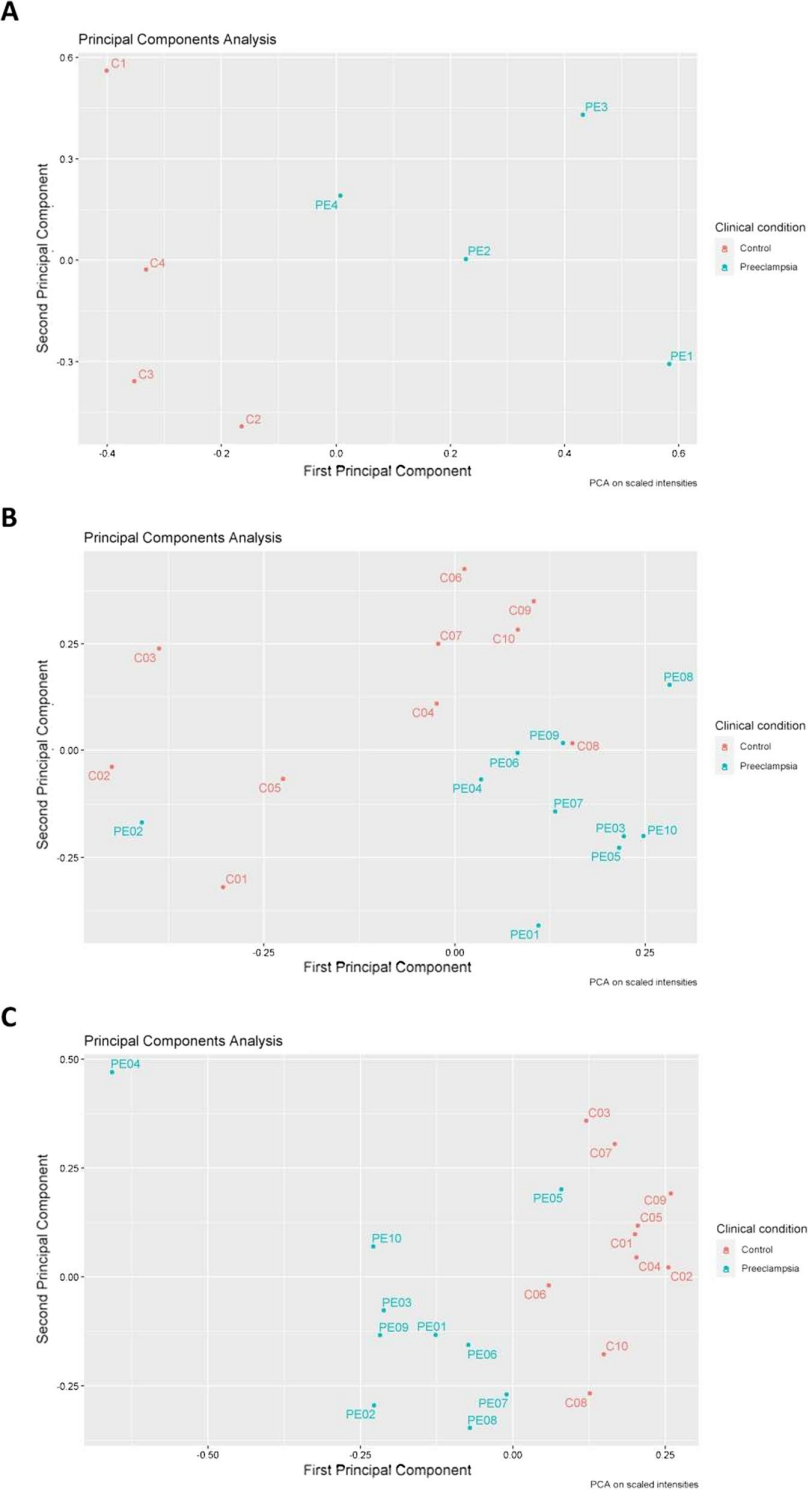
Unsupervised principal component analysis (PCA) results from multiple reaction monitoring data. Note sample clustering of control and pre-eclamptic conditions for sample collections C1 (**A**), C2 (**B**) and C3 (**C**). Note that PCA revealed as outliers samples e.g., C1, C8 and PE2 in sample collection C2, C8 in sample collection C3, which were discarded for quantitative proteomic analysis according to sample exclusion criterion (see “Materials and methods”)

In the present study, we explored the potential of extracellular vesicles, and particularly exosomes, as a source of biomarkers associated with pre-eclampsia. Exosomes are characterized by their size (50–150 nm) and by the presence of specific markers (e.g., CD9, CD63, and CD81) indicative of their endosomal origin, and their composition (cargo) reflects the cell or tissue from which they originate, as well as its physiological state [18]. It has been clearly demonstrated that the placenta and other pregnancy-associated tissues secrete exosomes into the extracellular space, including the blood [19, 47–49]. Likewise, there is increasing evidence that secreted exosomes interact with spatially distant cells and tissues, modulating multiple biological processes essential for the progress of pregnancy [21, 24, 50–53]. Although there is no standard reference method for the purification of extracellular vesicles, and particularly exosomes [54], different studies describe size-exclusion chromatography (SEC) as the method of choice with regards to the presence of contaminant soluble proteins, which provides highly pure isolates compared with other enrichment techniques [55–57]. This feature, combined with its simplicity and moderate cost, makes SEC the best option for efficient exosome isolation from plasma/serum. Our data show that the use of SEC of serum from women with healthy or pre-eclamptic pregnancies provides high-quality exosome samples, both from a morphological perspective (shape, size) and at the level of molecular composition. In this line, exosome preparations that did not contain a significant number of robust exosomal markers (e.g., CD9, CD63, CD81) [37], were excluded from further analysis. Unsupervised PCA results from MRM data revealed clear clustering of control and pre-eclamptic conditions in the 3 sample collections and highlighted several poor exosomal samples as outliers, which were discarded according to sample exclusion criteria (Fig. 3). Finally, we confirmed the presence of pregnancy-associated marker proteins (ALPP, PZP) by shotgun proteomics analysis, confirming that a significant although scarcely quantifiable percentage of the purified exosomes originated from organs and tissues associated with pregnancy (e.g., placenta, decidua).

Quantitative label-free proteomic analysis detected statistically-significant changes in the three studied sample collections. The overlap between the sets of differentially-regulated proteins reached 40%, despite the methodological differences and the different pregnancy stages in which they were collected (end of 2nd trimester *versus* delivery). Furthermore, 40% (4 out of 10) regulated proteins in sample collection C1 (obtained at delivery) showed the same trend as the other sample collections obtained at delivery. One of these proteins was versican, a proteoglycan that is part of the glycocalyx. Endothelial dysfunction leads to alterations in glycocalyx composition, with the consequent release of its constituents into the blood; these events have been reported in pre-eclampsia [58]. In addition to versican, our results confirm previous analyses describing increased levels (EOPE > C, C3) for another glycocalyx-associated protein, biglycan (BGN) [59]. Finally, the finding of other proteoglycans differentially regulated in C2 and/or C3, such as SDC and proteoglycan 4 (PRG4), suggest a close relationship between the glycocalyx, endothelial dysfunction and pre-eclampsia. Two of the differentially-regulated proteins common to the three sample collections, PZP and RELN inhibit the aggregation of misfolded proteins, and showed the same differential regulation trend (EOPE < C). The reduced expression of PZP has been proposed to be associated with protein aggregate accumulation in pre-eclampsia [60].

Remarkably, more than 30% of the proteins described as differentially regulated were shared between sample collections C2 (weeks 25–27) and C3 (delivery), although in approximately half of the results the direction of the quantitative change (EOPE > C or EOPE < C) was reversed. This panel of proteins includes 10 cytoskeletal keratins and desmoplakin (DSP), which have been described as constituents of desmosomes together with other proteins of the plakin family such as plakophilin-1 (PKP1) and -3 (PKP3), and envoplakin (EVPL) [61]. We found that the pre-eclampsia-associated downregulation (EOPE < C) found in C2 (weeks 25–27) of these proteins reverted to increased protein abundance (EOPE > C) at delivery (C3). As structural elements of desmosomes, plakins and keratins play important roles in cell development and migration, and pre-eclampsia-associated changes might impair the invasiveness of trophoblast cells [62]. Recently, Garrido-Gomez et al. [63] described that corneodesmosin, a major component of desmosomes, is highly upregulated in severe pre-eclampsia. Other differentially-regulated proteins described in the present study that have been previously associated with pre-eclampsia include tenascin (TNC) [64] and apolipoprotein A1 (APOA1) [65], among others. However, although some of the proteins currently used in clinical practice as markers of pre-eclampsia, including ENG or VEGFR, were identified in some sample collections (C3), they could not be reliably quantified.

To our knowledge, only two systematic quantitative analyses on exosomes purified from control or pre-eclamptic human plasma have been published to date [66, 67], and only one of them used methods based on quantitative proteomics [67]. Given the lack of similar studies that can be used to confirm our results, we compared them with global quantitative proteomic analyses performed on serum or plasma samples, without a prior exosome purification step. We used a very refined list of 29 protein markers detected in serum or plasma and previously described as associated with pre-eclampsia in 32 independent studies [17]. We found a total of 9 common markers between these studies and our results from samples collected at delivery. Of them, 6 markers, apolipoprotein E (APOE), AMBP, serotransferrin (TF), PZP, inter-alpha-trypsin inhibitor heavy chain H4 (ITIH4) and ficolin-2 (FCN2) showed similar regulation patterns (EOPE > C or EOPE < C), while 3 markers diverged: α-2-macroglobulin (A2M), fibronectin (FN1) and apolipoprotein B-100 (APOB). As placenta secretes extracellular vesicles into the milieu [18, 19, 23, 47, 48, 68], we thought it interesting to compare the results of our study with the biomarkers described from placental samples in a total of 23 independent studies [17], but we failed to find any matching protein. It is possible that the analysis of the entire placental proteome is not sufficiently sensitive to detect and quantify placental proteins that are transported in exosomes; alternatively, it is possible that the exosomes come mostly from tissues related to pregnancy but not from placenta per se.

Finally, we examined the potential effects of exosomes on the physiological state of cells and, more specifically, if they could alter the qualitative and quantitative composition of the cellular proteome. Previously published studies have shown that transfer of syncytiotrophoblast-derived extracellular vesicles to human endothelial cells is a fast and efficient process [69]. We performed a series of in vitro experiments, adding purified exosomes of different origin (from C/EOPE conditions) to two different cell types that are models of tissues relevant in pregnancy, namely, placenta (BeWo cells) and endothelium (HUVECs). We paid special attention to the amount of exosomes added to the cell cultures. A recently published study investigating vascular dysfunction used up to 100 µg of purified exosomes [22], which in our opinion is clearly unbalanced in relation to the number of target cells (corresponding approximately to 10–15 µg of proteome) and could skew the results. Our data show that the addition of a reduced amount (2 µg) of exosomes purified from pre-eclamptic *versus* normotensive sera significantly alters the composition of the BeWo cell proteome (human choriocarcinoma), increasing the expression levels of 56 proteins. Upregulated proteins were linked to relevant biological processes in pre-eclampsia such as hypoxia (e.g., hypoxia up-regulated protein 1) and protein stabilization (e.g., heat shock protein 90-alpha). Furthermore, the presence of upregulated proteins related to replication, transcription and translation processes suggests that cells respond to interaction with pre-eclamptic exosomes by activating these processes (Additional file 12: Table S8). A similar experiment performed on HUVECs did not yield conclusive results (data not shown).

## Supplementary Information

**Additional file 1: Figure S1.** Scheme of the experimental flow used in the proteomic analysis of exosomes purified from clinical serum samples (control / EOPE).

**Additional file 2: Figure S2a–c**. Boxplots of differences in estimated abundance in pre-eclampsia (PE) and control (C) samples. Only proteins with q < 0.1 are displayed. Sums of areas are in standardized scale so that they can be compared across proteins. A: results corresponding to differentially-regulated proteins (PE > C or PE < C) in sample collection C1 (at delivery); B: results corresponding to diferentially-regulated proteins (PE < C or PE < C) in C2 (w25-27); C: results corresponding to PZP (PE < C) in sample collection C4.

**Additional file 3: Table S1**. Summary of serum control and pre-eclampsia samples used in this study.

**Additional file 4: Table S2.** MRM transition list used in the targeted proteomic analysis of the 66 proteins detected with at least two proteotypic peptides (except for syndecan from C2 sample collection).

**Additional file 5: Table S3a**. Total number of peptides (FDR < 1%, calculated at peptide level) and proteins identified in the quantitative proteomic analysis of exosomes purified from collection C1 (at delivery) samples.

**Additional file 6: Table S3b**. Total number of peptides (FDR < 1%, calculated at peptide level) and proteins identified in the quantitative proteomic analysis of exosomes purified from collection C2 (weeks 25–27) samples.

**Additional file 7: Table S3c**. Total number of peptides (FDR < 1%, calculated at peptide level) and proteins identified in the quantitative proteomic analysis of exosomes purified from collection C3 (at delivery) samples.

**Additional file 8: Table S4**. Functional analysis of proteins identified in sample collections C1–C3. Information related to tissue specificity, developmental stage and involvement in disease was obtained from public repositories.

**Additional file 9: Table S5**. Summary of differentially-regulated proteins found in the label-free based quantitative proteomic analysis of control and pre-eclamptic serum exosomes from sample collections C1–C3. Only statistically significant differences (q < 0.1) are shown.

**Additional file 10: Table S6**. Comparison of proteomic quantitative results of proteins shared between sample collections C1–C3.

**Additional file 11: Table S7**. Functional analysis of the differentially-regulated proteins described for sample collections C1–C3.

**Additional file 12: Table S8**. Statistical analysis of the MRM results corresponding to the 66 proteins detected with at least two proteotypic peptides (except for syndecan from C2 sample collection).

**Additional file 13: Table S9**. Label-free-based quantitative proteomic analysis results obtained at in vitro stimulation assays of BeWo cells using exosomes isolated from serum.

## Data Availability

The datasets generated and/or analyzed during the current study are available in the ProteomeXchange Consortium via the PRIDE partner repository [70] with the dataset identifier PXD025308 and https://doi.org/10.6019/PXD025308.

## Abbreviations

EOPE: Early-onset pre-eclampsia
FDR: False discovery rate
HUVEC: Human umbical vein endothelial cells
LC–MS/MS: Liquid chromatography coupled to tandem mass spectrometry
MRM: Multiple reaction monitoring
NTA: Nanoparticle tracking analysis
PCA: Principal component analysis
PlGF: Placental growth factor
SEC: Size-exclusion chromatography
sEV: Small extracellular vesicles
TEM: Transmission electron microscopy
TGF-β1: Transforming growth factor beta 1
VEGF: Vascular endothelial growth factor
VEGFR1: Vascular endothelial growth factor receptor
