# Supplementary material for: Quantitative proteomic analysis of serum-purified exosomes identifies putative pre-eclampsia-associated biomarkers
Source: Clin Proteomics. 2022 Feb 10;19:5. doi: 10.1186/s12014-022-09342-4 (PMC8903615; doi:10.1186/s12014-022-09342-4)
Supplement: Supplementary file 1 — Additional file 1: Figure S1. Scheme of the experimental flow used in the proteomic analysis of exosomes purified from clinical serum samples (control / EOPE). [file 12014_2022_9342_MOESM1_ESM.pdf]

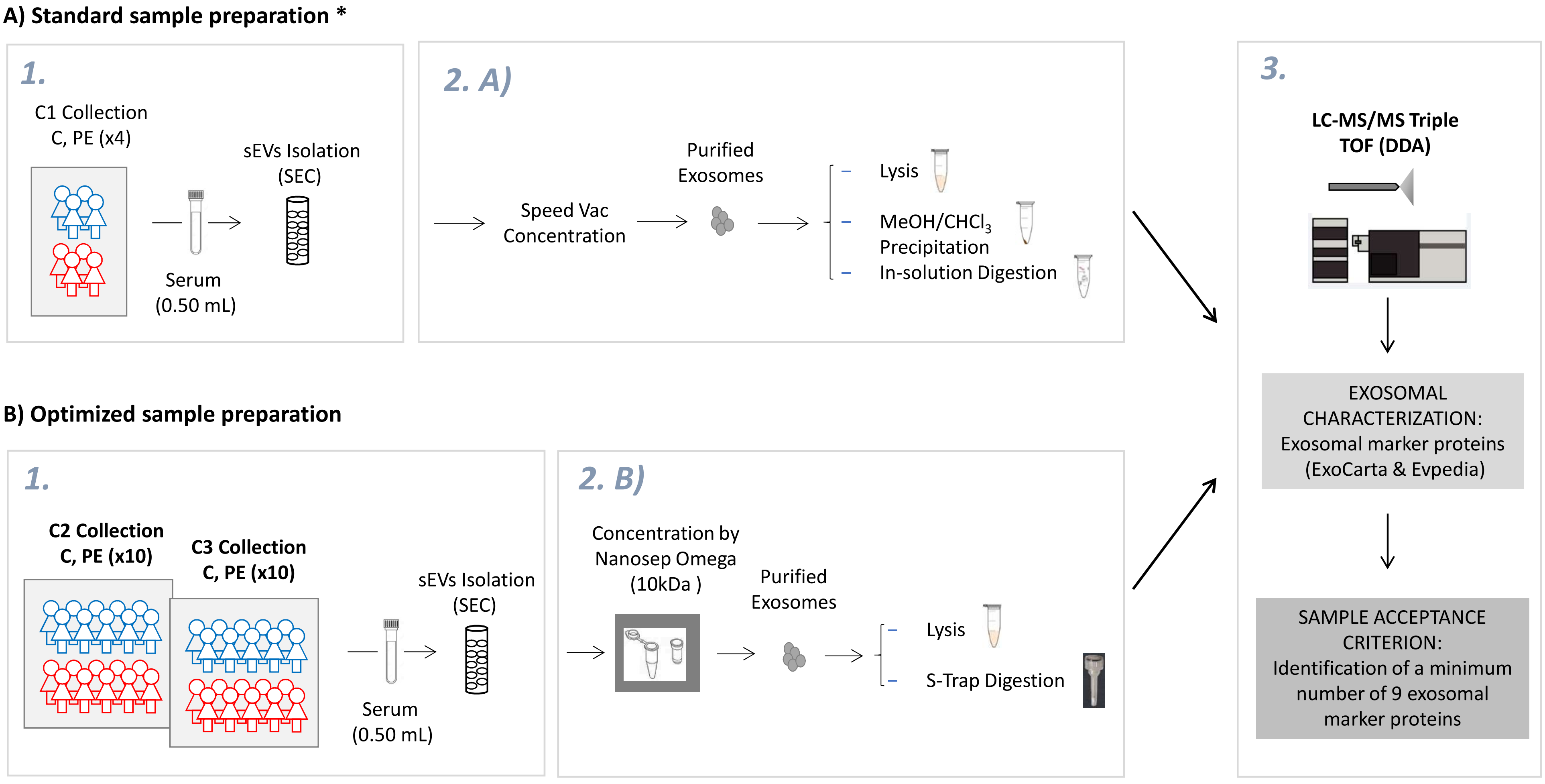

* C4 collection (11 control and 11 preeclampsia samples), only used for PZP validation by MRM, also followed the standard sample preparation
