# Supplementary figures and images for: Quantitative proteomic analysis of serum-purified exosomes identifies putative pre-eclampsia-associated biomarkers
Source: Clin Proteomics. 2022 Feb 10;19:5. doi: 10.1186/s12014-022-09342-4 (PMC8903615; doi:10.1186/s12014-022-09342-4)

A

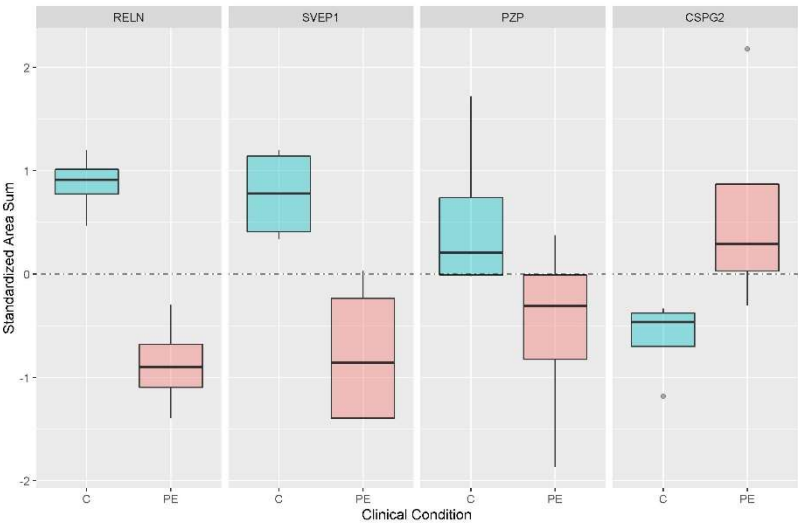

B

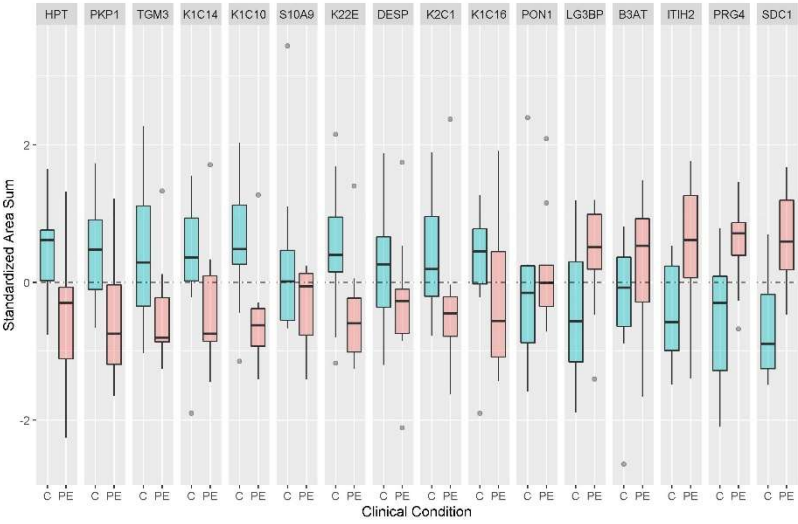

C

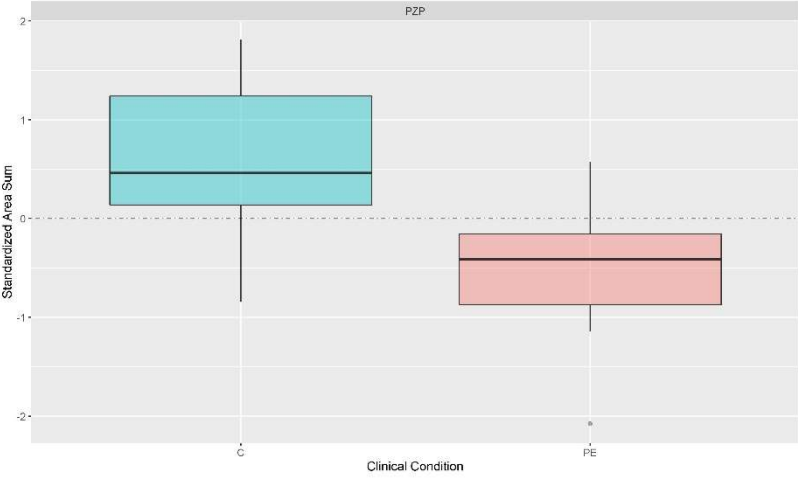

Supplement: Supplementary file 2 — Additional file 2: Figure S2a–c. Boxplots of differences in estimated abundance in pre-eclampsia (PE) and control (C) samples. Only proteins with q < 0.1 are displayed. Sums of areas are in standardized scale so that they can be compared across proteins. A: results corresponding to differentially-regulated proteins (PE > C or PE < C) in sample collection C1 (at delivery); B: results corresponding to diferentially-regulated proteins (PE < C or PE < C) in C2 (w25-27); C: results corresponding to PZP (PE < C) in sample collection C4. [file 12014_2022_9342_MOESM2_ESM.pdf]
